# Prediction of homologous recombination deficiency from routine histology with attention-based multiple instance learning in nine different tumor types

**DOI:** 10.1186/s12915-024-02022-9

**Published:** 2024-10-08

**Authors:** Chiara Maria Lavinia Loeffler, Omar S. M. El Nahhas, Hannah Sophie Muti, Zunamys I. Carrero, Tobias Seibel, Marko van Treeck, Didem Cifci, Marco Gustav, Kevin Bretz, Nadine T. Gaisa, Kjong-Van Lehmann, Alexandra Leary, Pier Selenica, Jorge S. Reis-Filho, Nadina Ortiz-Bruechle, Jakob Nikolas Kather

**Affiliations:** 1 Department of Medicine III, University Hospital RWTH Aachen, Aachen, Germany; 2 Else Kroener Fresenius Center for Digital Health, Medical Faculty Carl Gustav Carus, Technical University Dresden, Dresden, Germany; 3 Department of Medicine I, Faculty of Medicine Carl Gustav Carus, University Hospitaland, Technische Universität Dresden , Dresden, Germany; 4 Department for Visceral, Thoracic and Vascular Surgery, University Hospital Carl Gustav Carus, Technical University Dresden, Dresden, Germany; 5 Pathology & Data Analytics, Leeds Institute of Medical Research at St James’s, University of Leeds, Leeds, UK; 6 Medical Oncology, National Center for Tumor Diseases (NCT), University Hospital Heidelberg, Heidelberg, Germany; 7 Institute of Pathology, University Hospital RWTH Aachen, Aachen, Germany; 8 Joint Research Center Computational Biomedicine, University Hospital RWTH Aachen, Aachen, Germany; 9 Center for Integrated Oncology, Aachen Bonn Cologne Duesseldorf (CIO ABCD), Duesseldorf, Germany; 10 Cancer Research Center Cologne-Essen, University Hospital Cologne, Cologne, Germany; 11 Gynecological Cancer Unit, Department of Medicine, Institut Gustave Roussy, Villejuif, France; 12 Experimental Pathology, Department of Pathology, Memorial Sloan Kettering Cancer Center, New York, NY USA

**Keywords:** Homologous recombination deficiency, Deep learning, DNA repair mechanism, Artificial intelligence, Mpathology, Pan-cancer study

## Abstract

**Background:**

Homologous recombination deficiency (HRD) is recognized as a pan-cancer predictive biomarker that potentially indicates who could benefit from treatment with PARP inhibitors (PARPi). Despite its clinical significance, HRD testing is highly complex. Here, we investigated in a proof-of-concept study whether Deep Learning (DL) can predict HRD status solely based on routine hematoxylin & eosin (H&E) histology images across nine different cancer types.

**Methods:**

We developed a deep learning pipeline with attention-weighted multiple instance learning (attMIL) to predict HRD status from histology images. As part of our approach, we calculated a genomic scar HRD score by combining loss of heterozygosity (LOH), telomeric allelic imbalance (TAI), and large-scale state transitions (LST) from whole genome sequencing (WGS) data of *n* = 5209 patients across two independent cohorts. The model’s effectiveness was evaluated using the area under the receiver operating characteristic curve (AUROC), focusing on its accuracy in predicting genomic HRD against a clinically recognized cutoff value.

**Results:**

Our study demonstrated the predictability of genomic HRD status in endometrial, pancreatic, and lung cancers reaching cross-validated AUROCs of 0.79, 0.58, and 0.66, respectively. These predictions generalized well to an external cohort, with AUROCs of 0.93, 0.81, and 0.73. Moreover, a breast cancer-trained image-based HRD classifier yielded an AUROC of 0.78 in the internal validation cohort and was able to predict HRD in endometrial, prostate, and pancreatic cancer with AUROCs of 0.87, 0.84, and 0.67, indicating that a shared HRD-like phenotype occurs across these tumor entities.

**Conclusions:**

This study establishes that HRD can be directly predicted from H&E slides using attMIL, demonstrating its applicability across nine different tumor types.

**Supplementary Information:**

The online version contains supplementary material available at 10.1186/s12915-024-02022-9.

## Background

Homologous recombination (HR) is a DNA repair mechanism that ensures genomic integrity after DNA double-strand breaks (DSBs), a common occurrence during the cell cycle [1]. The lack of this process, referred to as homologous recombination deficiency (HRD), results in defective DNA break repair leading to increased somatic copy number alterations and genomic instability, thereby driving malignant transformation and cancer development [2]. According to the genomic definition of HRD, its prevalence varies among different tumor types, ranging from 0% in thymoma or thyroid cancer to as high as 70% in ovarian cancer [3]. Within the biological and clinical context of HRD, poly(ADP-ribose)-polymerase (PARP) plays an essential role in repairing single-strand DNA breaks (SSDBs) via base excision repair and by acting as a key compensatory mechanism within this process [4]. Under proficient HR conditions, PARP inhibition leads to the accumulation of unrepaired SSDBs, which subsequently convert into DSBs. HR can repair these DSBs, thus preserving genomic integrity and cell viability. Conversely, in HRD tumors, PARP inhibition induces DSBs that remain unrepaired, resulting in direct cytotoxicity. This interplay is the basis of synthetic lethality, eliciting the importance of HRD as a biomarker that can aid in identifying patients who might benefit from PARP inhibitor (PARPi) therapy across several tumor types, such as breast, ovarian, prostate, and pancreatic cancers [5–8]. Moreover, clinical trials have underscored the significance of PARPi in improving disease-free survival by increasing platinum sensitivity, notably in ovarian and breast cancer, as well as in other tumor types [4, 9, 10]. Nevertheless, the benefits of PARPi therapy are significantly constrained by the challenges in diagnosing HRD, given the diverse and varying comprehensiveness of the current HRD assessment strategies.

**Fig. 1:**
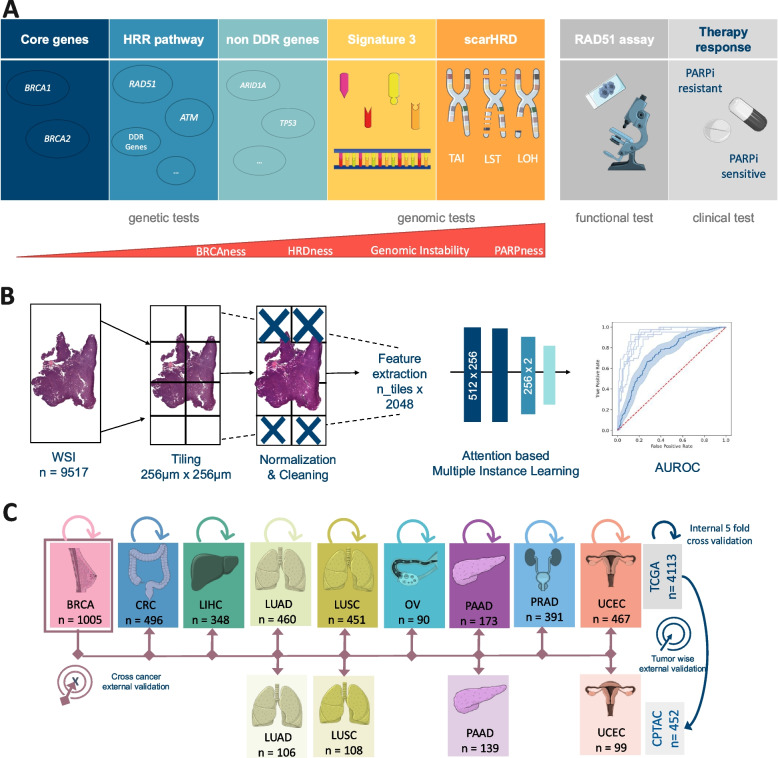
Experimental design and study overview. **A** Overview of the different Homologous Recombination Deficiency (HRD) scores, their content, and assessment methods. **B** Workflow of our deep learning (DL) pipeline. A total of *n* = 9517 whole slide images (WSI) were processed and trained with an attention-based multiple instance learning (attMIL) approach. The statistical endpoint was the Area under the receiving operating curve (AUROC). **C** Study design for the three main experiments (internal fivefold cross-validation, tumor-wise external validation, and cross-cancer external validation) conducted and cohort overview for patients and tumor types included from The Cancer Genome Atlas (TCGA, *n* = 4113 patients) and Clinical Proteomic Tumor Analysis Consortium (CPTAC, *n* = 452 patients). Abbreviations: BRCA, breast invasive carcinoma; CRC, colorectal cancer; LIHC, liver hepatocellular carcinoma; LUAD, lung adenocarcinoma; LUSC, lung squamous cell carcinoma; OV, ovarian serous cystadenocarcinoma; PAAD, pancreatic adenocarcinoma; PRAD, prostate adenocarcinoma; UCEC, uterine corpus endometrial carcinoma; HRR, Homologous recombination repair. This figure was partly generated using Servier Medical Art, provided by Servier, licensed under a Creative Commons Attribution 3.0 unported licence

These assessments can be broadly categorized into three main groups: genetic, genomic, and functional testing (Fig. 1A). Among these, genetic HRD tests primarily focus on identifying oncogenic germline mutations in the Breast Cancer genes 1 and 2 (*BRCA1/2*), which are commonly observed in breast, prostate, and pancreatic cancer [11, 12]. At the same time, relying solely on *BRCA1/2*-related mutations to diagnose HRD risks overlooking cases [13], particularly because HRD can also arise from other mechanisms, such as epigenetic modifications, as well as germline and somatic mutations in genes associated with or outside the HRR pathway [14]. For instance, in ovarian cancer, up to 10% of patients demonstrate HRD without *BRCA1*/2 mutations [15]. Another key indicator of HRD is genomic instability, which is evident via patterns in structural variants such as loss of heterozygosity (LOH), telomeric allelic imbalance (TAI), and large-scale state transitions (LST) [11, 16]. When these alterations are widespread across the genome, they contribute to a quantifiable genomic instability score (GIS) [13, 17]. Genomic HRD tests harness whole genome sequencing and single nucleotide polymorphism (SNP) array data to identify LOH, TAI, and LST markers and have proven to be effective in predicting the benefits of PARPi therapy in randomized clinical trials [18–20]. Biologically, this method provides a comprehensive assessment of genomic instability due to HRD. Hence, for this study, we mainly focused on the genomic HRD test (Fig. 1A). However, due to its complexity, GIS has yet to be implemented in routine diagnostics in clinical workflows [12, 13, 21]. Therefore, the gold standard for identifying what is known as the genomic “scar” of HRD, currently comprises the combination of different algorithms such as scarHRD, HRDetect, and CHORD [22–24]. It is important to mention that in addition to these genetic consequences, HRD can also result in functional repercussions, which can be assessed through non-DNA-based functional tests, such as the *RAD51* focus formation assays [25–27]. The U.S. Food and Drug Administration (FDA) has approved HRD tests, like FoundationOne CDx (Foundation Medicine, Inc., Cambridge, MA) and myChoice CDx (Myriad Genetics Laboratories, Inc., Salt Lake City, UT), which utilize a combination of BRCA1/2 mutations and LOH or GIS for diagnostic results [11, 12, 28]. However, the absence of uniform pan-cancer cut-off values for categorizing HRD cases remains a challenge in HRD testing [29, 30], often leading to suboptimal patient classification. This underscores the need for more clinical research to define cancer-specific HRD cut-offs. Within the last decade, the field of artificial intelligence (AI) has yielded powerful methods such as deep learning (DL), which allow features to be quantitatively extracted from whole slide images (WSIs). These DL tools have enabled the detection of genetic alterations directly from histopathological image data [31–33]. Some examples include the prediction of phenotypic changes attributed to single mutations [34, 35], as well as DNA instability mechanisms such as microsatellite instability (MSI), from routine histopathology WSIs stained with hematoxylin and eosin (H&E) [36, 37]. Today, several DL models have received regulatory approval and are available for diagnostic use in Europe and the USA [38]. Although previous studies have shown promising results in predicting HRD from WSI in cancers such as breast and ovarian, they have not investigated the extent to which HRD might be predictable as a pan-cancer biomarker in multiple cancer types [39, 40]. For this reason, we propose that tumor phenotypes, visible in histological WSIs, may also indicate genomic HRD status, which can be identified using DL models.

In this study, we developed a proof-of-concept DL model based on “attention-based Multiple Instance Learning” (attMIL) and weakly supervised training using no spatial labels or manual annotations [33], for the prediction of HRD status directly from H&E WSIs. HRD ground truth was obtained through the use of scarHRD, a comprehensive method which assesses a variety of genomic changes [3, 22] in order to calculate an HRD score (Fig. 1B), as well as a widely recognized clinical cut-off point as our benchmark to address the complex and somewhat discordant landscape of current HRD testing [3, 41]. We then trained and evaluated the DL classifiers via cross-validation within a large cohort of *n* = 3881 patients from The Cancer Genome Atlas (TCGA), across nine different types of solid tumors. The models were then externally validated with an independent validation dataset (*n* = 452) in a tumor-wise and cross-cancer experimental approach (Fig. 1C) among four various cancer types. Taken together, our experimental results provide direct evidence that genomic HRD can be detected with DL from routine histology across different tumor types. This method may offer a new diagnostic approach that meets the clinical need for a cost-effective, rapid, and universally applicable HRD test, improving patient stratification and treatment options.

## Methods

### Data acquisition

Initially, data from 4735 patients for nine tumor types within The Cancer Genome Atlas (TCGA), and 474 patients from four tumor types from the Clinical Proteomic Tumor Analysis Consortium (CPTAC; Fig. 1C) were retrieved through https://www.cbioportal.org/. The tumor types included in this study were breast cancer (TCGA-BRCA, *n* = 1058), colorectal cancer (TCGA-CRC, *n* = 580), hepatocellular carcinoma (TCGA-LIHC, *n* = 364), lung adenocarcinoma (TCGA-LUAD, *n* = 536, CPTAC-LUAD, *n* = 111), lung squamous cell carcinoma (TCGA-LUSC, *n* = 497; CPTAC-LUSC, *n* = 109), ovarian serous cystadenocarcinoma (TCGA-OV, *n* = 520), pancreatic adenocarcinoma (TCGA-PAAD, *n* = 177; CPTAC-PAAD, *n* = 153), prostate adenocarcinoma (TCGA-PRAD, *n* = 488), and uterine corpus endometrial carcinoma (TCGA-UCEC, *n* = 515; CPTAC-UCEC, *n* = 101; Additional File 1: Fig. 1A, B). Image data and corresponding clinical data were available in TCGA-BRCA for *n* = 1005, TCGA-CRC for *n* = 496, TCGA-LIHC for *n* = 348, TCGA-LUAD for *n* = 460, CPTAC-LUAD for *n* = 106, TCGA-LUSC for *n* = 451, CPTAC-LUSC for *n* = 108, TCGA-OV for = 90, TCGA-PAAD for *n* = 173, CPTAC-PAAD for *n* = 139, TCGA-PRAD for *n* = 391, TCGA-UCEC for *n* = 467, and CPTAC-UCEC for *n* = 99, thus resulting in a total *n* = 4333 (TCGA *n* = 3881, CPTAC *n* = 452, Fig. 1C, Additional File 1: Fig. 1A, B) patients. Data from TCGA-BRCA corresponding to Riaz et al.’s study [42], was retrieved for additional experiments on *BRCA1/2* mutational status. Estrogen receptor data for the subgroup analysis were available only for *n* = 661 patients in the TCGA-BRCA cohort.

### Image preprocessing

Formalin-fixed, paraffin-embedded (FFPE) tissue slides were downloaded for the TCGA cohorts from the GDC Portal (https://portal.gdc.cancer.gov/), and frozen tissue slides for the CPTAC cohort from The Cancer Imaging Archive (https://www.cancerimagingarchive.net/). Images were first tessellated into patches with an edge length of 256 µm and a resolution of 224 × 224 pixels. Secondly, the patches for each cohort were color normalized using the Macenko spectral matching technique [43] to enforce a standardized color distribution across the cohorts. Prediction models were trained using our in-house open-source DL pipeline “marugoto,” accessible at https://github.com/KatherLab/marugoto. The pipeline consists of a self-supervised learning (SSL) model, leveraging ResNet50, a deep convolutional neural network pretrained with ImageNet weights and fine-tuned on a pan-cancer dataset of approximately 32,000 WSIs. The model extracts a 2048-dimensional feature vector for each patch per patient [44]. To obtain patient-level predictions, 512 × 2048 feature matrices, referred to as MIL bags, were constructed. This is done by concatenating 512 feature vectors randomly selected for each patient. These matrices were fed into an attMIL framework with the following architectures: 512 × 256 and 256 × 2 (Fig. 1B) [45, 46]. To ensure the robustness of our findings, we also performed the same experiments using another pretrained vision transformers encoder called UNI [47], https://github.com/mahmoodlab/UNI), followed by a transformer-based multi-head self-attention DL-model as previously already published [48] under: https://github.com/KatherLab/marugoto/tree/transformer. We refer to this second method as the transformer-based DL model. The transformer-based DL-model was trained under the same conditions to allow for a comparison of results.

### Calculation of HRD scores

For patient-wise calculation of a genomic HRD score, single nucleotide polymorphism (SNP) data, generated by the allele-specific copy number analysis of tumors (ASCAT) algorithm were downloaded from the Genomic Data Commons (GDC) Portal: https://portal.gdc.cancer.gov/ (accessed 06/15/2022) for all cohorts. The HRD score was calculated using scarHRD (https://github.com/sztup/scarHRD), as described in previous studies [3, 22]. ScarHRD determines HRD using whole genome sequencing data in the form of SNP arrays to calculate the three subscores LOH, LST, and TAI. The sum of these subscores composes the patient-wise HRD score (Fig. 1A) [49]. The cut-off values for the different subscores were previously defined by Abkevich et al. for LOH, Popova et al. for LST, and Birkbak et al. for TAI [18–20]. By summing up the LOH, LST, and TAI scores, patients can be binarized into HRD high (HRD-H) and HRD low (HRD-L) groups at a cut-off of 42, which has been also used in other studies and clinical trials [3, 30, 41, 50], as well as in our analysis. For CPTAC, the respective data were only available for the CPTAC-3 cohort (Additional File 1: Fig. 1A, B).

### Experimental design

In our study, we performed three main experiments (Fig. 1B). To assess the baseline predictability of HRD from routine histology, we first trained a classifier using five-fold-cross-validation within each of the nine tumor entities mentioned above in the TCGA cohorts (internal validation). This was achieved by randomly splitting each cohort at the patient level, creating non-overlapping training and test sets for model training. The splitting ratio was 60:20:20 for the training, validation, and test splits in all the experiments. Internal validation was performed in a fivefold cross-validated design, so that no data leakage from the training to the test set occurred. This process was repeated individually for each cancer type in the TCGA cohorts. A weighted cross-entropy loss function was used to assist the model with the imbalanced dataset. Secondly, we deployed the five-fold-cross models trained in the first experiments on the same tumor type from the CPTAC cohorts as an external validation. By utilizing this approach, we circumvented any potential claims of selecting the model with the highest AUROC in the external validation. Finally, we trained an HRD classifier on the TCGA-BRCA cohort, which had the highest number of patients, and deployed it on all other TCGA cohorts (CRC, LIHC, LUAD, LUSC, PRAD, PAAD, OV, UCEC) as well as on all CPTAC cohorts (LUAD, LUSC, PAAD, UCEC). In our study, we aimed to evaluate the performance of the models using the AUROC, which is commonly used for assessing the accuracy of binary classification tasks. Our primary statistical endpoint was the AUROC ± 95% confidence interval (CI) and Area under the precision-recall curve (AUPRC; Additional File 2: Table 1). To further assess the performance of each model, we used a two-sided *t*-test to compare the patient-level prediction scores between the HRD-H and HRD-L patient groups as defined by the ground truth and reported the p-values, assuming a significance level of < 0.05 as statistically significant, without correction for multiple testing (Additional File 2: Table 1). As a final step to obtain a more in-depth understanding of the TCGA-BRCA cohort, we uploaded our custom HRD-H and HRD-L ground truths and predicted subgroups in cBioPortal to examine the characteristics of these patients in the TCGA-BRCA PanCancer Atlas cohorts.

### Explainability

To visualize our model’s output, we created high-resolution heatmaps displaying the spatial distribution of the attention and prediction scores on the original WSI. We extracted the image feature vectors for 32 × 32 pixels from the WSI using the RetCCL convolutional neural network. Attention and classification scores were calculated for each image region and normalized across the patient cohort. Based on these scores, color heatmaps were generated for each patient. Red color indicates high attention or a positive classification and blue color indicates low attention or a negative classification. To ensure the interpretability of the underlying morphology alongside with the attention and classification scores, we reconstructed the final attention and classification heatmaps separately by blending the raw color heatmaps with the image features. This approach allows us to interpret the output of our model in a way that is easy to understand and provides insight into the underlying morphology of the tumor.

## Results

### HRD is predictable from histology with attmil

**Fig. 2:**
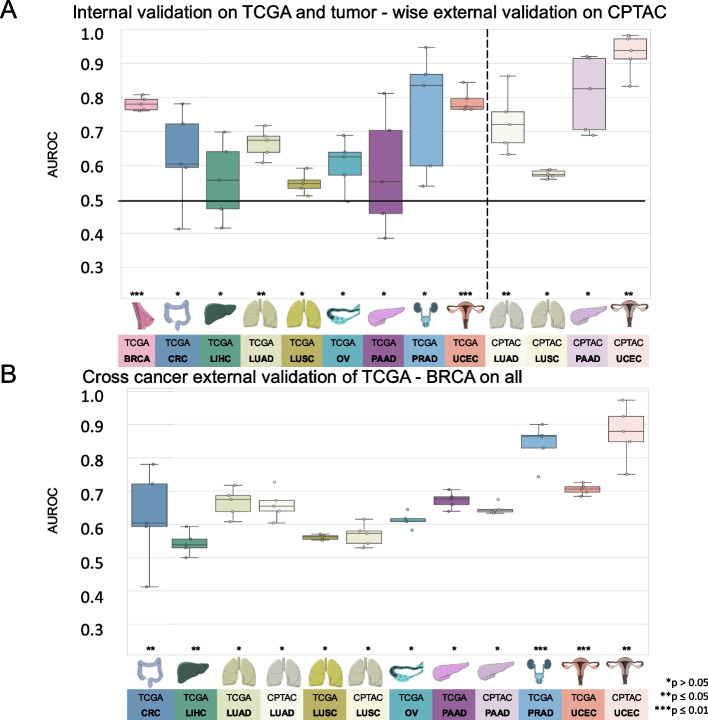
Comparison of the area under the receiving operating curve (AUROC) for internal and tumor-wise external validation experiment models. Boxplot displaying the distribution of the AUROC and *p*-value (*p > 0.05; **p ≤ 0.05; ***p ≤ 0.01) for **A** internal fivefold cross-validation experiment of The Cancer Genome Atlas (TCGA) and tumor-wise external validation on the Clinical Proteomic Tumor Analysis Consortium (CPTAC); **B** AUROCs for the cross-cancer external validation experiment of the TCGA breast invasive carcinoma cohort (TCGA-BRCA) on the TCGA and CPTAC cohort. The horizontal line indicates the median, whereas each box represents the interquartile range (IQR) between the first and third quartiles. The whiskers extend from the box to the minimum and maximum values, considering 1.5 times the IQR. Abbreviations: BRCA, breast invasive carcinoma; CRC, colorectal cancer; LIHC, liver hepatocellular carcinoma; LUAD, lung adenocarcinoma; LUSC, lung squamous cell carcinoma; OV, ovarian serous cystadenocarcinoma; PAAD, pancreatic adenocarcinoma; PRAD, prostate adenocarcinoma; UCEC, uterine corpus endometrial carcinoma

First, we investigated whether DL could predict HRD status from H&E-stained slides within nine different cancer types from the TCGA cohort. We used cross-validation on the patient level to train and test an attMIL-based DL model within each cohort. According to our dataset, the incidence of HRD ranged from 3% in PRAD and up to 63% in OV (Additional File 1: Fig. 1C). We found that in five out of the nine cancer types, the mean prediction AUROC was above 0.6, and the 95% CI of the fold-wise HRD prediction AUROCs remained above the null hypothesis of 0.5. Among these, HRD prediction reached statistical significance, with a *p*-value below 0.05 for three cancer types: UCEC (AUROC 0.79 ± 0.04, *p* = 0.0008), BRCA (AUROC 0.78 ± 0.02, *p* < 0.0001) and LUAD (AUROC 0.66 ± 0.05, *p* = 0.02; Fig. 2A). The AUPRC values are reported in Additional File 2: Table 1. The prediction of HRD was not possible in LUSC, LIHC, as their AUROCs did not exceed the baseline (0.55 ± 0.04, 0.56 ± 0.14) (Additional File 3: Fig. 2 A–I, Additional File 2: Table 1). For the tumor types PAAD, OV, and PRAD, the AUROCs ranged from 0.58 ± 0.22 to 0.6 ± 0.09 to 0.76 ± 0.22. Taken together, these data demonstrate that DL can predict HRD status from histology images alone for several tumor types.

### HRD is predictable from H&E staining with attmil in an independent test set

A key part of successfully developing deep learning models is to externally validate them using WSIs from patient cohorts that are completely separate from the training set [51]. Hence, for our external validation experiments, we deployed the models obtained from the cross-validation training on TCGA to analyze cohorts from the CPTAC dataset corresponding to the same cancer type. External validation cohorts in CPTAC were available for UCEC, PAAD, LUAD, and LUSC. In these external validation experiments, the prediction performance was better than that in the internal validation experiments. Once again, the best performance was obtained in UCEC, with an AUROC of 0.93 ± 0.07, *p* = 0.01. In LUAD the performance improved, yielding an AUROC of 0.73 ± 0.11 and a significant *p*-value of 0.03. In the case of PAAD, where the internal validation was unsuccessful (internal validation AUROC 0.58 ± 0.22), the external validation resulted in an improved AUROC reaching 0.81 ± 0.14, albeit with a *p*-value of 0.07. Meanwhile, in LUSC, no improvement in performance was observed in the external validation set compared to the internal training set (AUROC 0.57 ± 0.01, *p* = 0.23, Fig. 2A, Additional File 3: Fig. 2 J–M). Together, these data show that DL-based classifiers for genomic HRD status generalize beyond the training cohort.

### HRD classifier trained on TCGA-BRCA detects HRD across various types of *cancer*

As our next step, we aimed to investigate whether HRD-related morphological features within a specific cancer type are able to predict HRD status in another cancer type. Thus, providing insight as to whether a shared set of morphological features across diverse cancer types could potentially allow a pan-cancer pathology-based prediction system for HRD status. To test this hypothesis, we applied our trained HRD classifiers in a cross-cancer experimental design. The HRD classification model was trained with the TCGA-BRCA cohort and deployed on all other cohorts obtained from the TCGA and CPTAC datasets. Surprisingly, the BRCA-based model was able to significantly predict genomic HRD from non-BRCA tissue in UCEC, PRAD, CRC, and LUAD. For three of those cohorts, the external deployment of a BRCA-based model resulted in higher prediction AUROCs than did the respective internal validation experiments, with AUROCs of 0.70 ± 0.02, *p* < 0.001 in TCGA-UCEC; 0.84 ± 0.07, *p* = 0.004 in TCGA-PRAD, 0.65 ± 0.03, *p* = 0.04 in TCGA-CRC and 0.87 ± 0.1, *p* = 0.05 in CPTAC-UCEC, respectively (Fig. 2B). For LUAD and OV, the AUROCs remained with 0.62 ± 0.03 for TCGA-LUAD, 0.66 ± 0.06 for CPTAC-LUAD and 0.61 ± 0.03 in TCGA-OV in a similar range to the internal validation results (Additional File 4: Fig. 3A–L). Together, these data show that a classifier trained on BRCA is able to predict HRD status from histology in other tumor types, suggesting a shared “HRD morphology.”

**Fig. 3:**
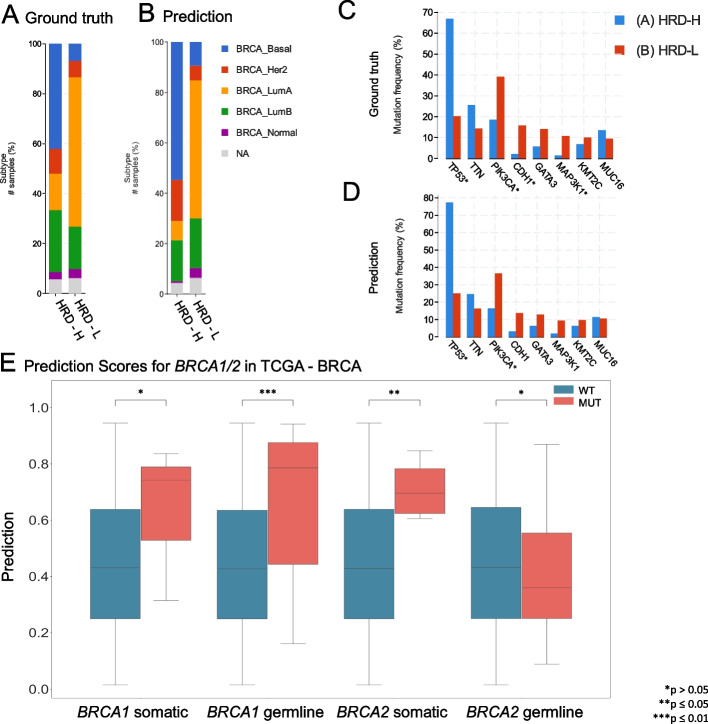
Molecular characterization of The Cancer Genome Atlas breast cancer (TCGA-BRCA) cohort. **A** Distribution of breast cancer subtypes for the homologous recombination deficiency high (HRD-H) and low (HRD-L) ground truth subgroups. **B** Distribution of the breast cancer subtypes for the HRD-H and HRD-L deep learning (DL) predicted subgroups. **C** Alteration frequency for several genes of the HRD-H and HRD-L ground truth subgroups. **D** Alteration frequency for several genes of the HRD-H and HRD-L within cohort internal results prediction subgroups. **E** Grouped boxplots comparing the homologous recombination deficiency high (HRD-H) prediction scores with the mutational status (mutated = MUT, wildtype = WT) for the somatic and germline alterations of the *BRCA1/2* genes. The central line represents the median value, while the box ranges between the first and third quartiles (IQR), and the whiskers extend to the lowest and highest values within 1.5 times the IQR. The *y*-axis represents the deep learning (DL) HRD-H prediction values. An independent *t*-test was performed to calculate the *p*-values (*p > 0.05; **p ≤ 0.05; ***p ≤ 0.01). This figure was created using https://www.cbioportal.org/ [59, 60]

To benchmark our results, we compared the performance of our attMIL with a transformer-based approach. In the internal cross-validation, the attMIL approach outperformed the transformer-based DL model in five out of nine experiments. In the tumor-wise external validation analysis the attMIL performed better in two out of four experiments. In the cross-cancer approach, the transformer-based approach outperformed the attMIL in six out of thirteen experiments. In summary, the transformer-based DL model yielded similar AUROCs compared to the attMIL approach, confirming the robustness of our initial findings. Detailed results of this comparison are provided in Additional File 2: Table 1.

#### Molecular and histomorphological characterization of TCGA-BRCA HRD-H and HRD-L patients

Finally, we investigated which molecular and morphological patterns were associated with the ground truth and DL-predicted genomic HRD status. In order to acquire a detailed analysis, we used the TCGA-BRCA cohort as it was the largest one available. We observed that in the HRD-H subgroup, 45% of the patients were classified as basal-like breast cancers, 11% as *HER2*-enriched, 15% as Luminal A, and 26% as Luminal B. In contrast, only 7% of the cases in the HRD-L subgroup were basal-like, 7% were *HER2*-enriched, 64% were Luminal A, and 18% were Luminal B (Fig. 3A) [52]. Within our predicted groups, we observed a similar distribution among the BRCA subtypes (Fig. 3B).

**Fig. 4:**
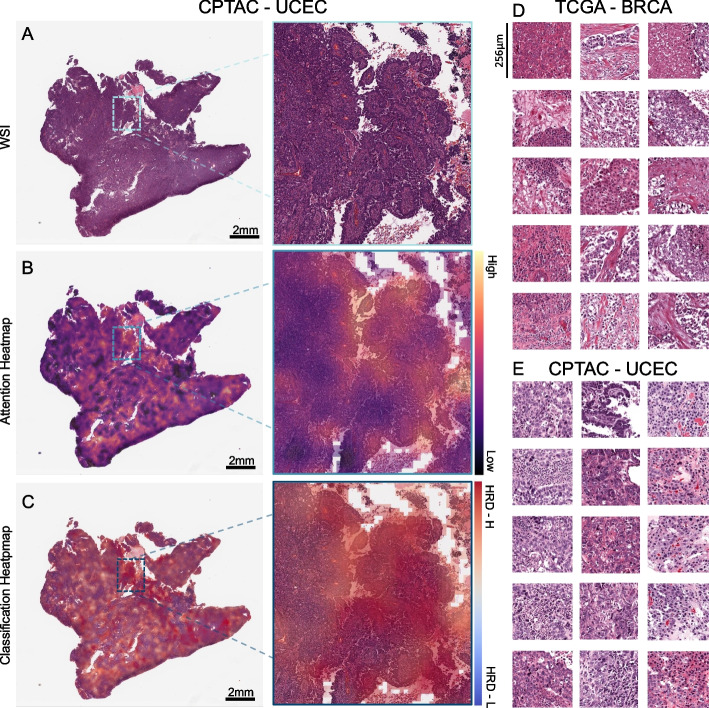
Visualization of predicted homologous recombination deficiency high (HRD-H) tumor samples. **A** Whole slide image (WSI) of an HRD-H predicted patient (ID: C3L-00358–21) from the Clinical Proteomic Tumor Analysis Consortium (CPTAC) uterine corpus endometrial carcinoma (UCEC) cohort with magnification. **B** Attention heatmap for the same patient with magnification. **C** Classification Heatmap for the same patient with magnification. **D** Top predicted tiles for top three homologous recombination deficiency high (HRD-H) patients in The Cancer Genome Atlas (TCGA) breast invasive carcinoma (BRCA). **E** Top predicted tiles for three HRD-H patients in the CPTAC-UCEC cohort

To confirm that our model predicts HRD based on the phenotypic differences between estrogen receptor-negative (ER −) and ER-positive (ER +) breast cancer samples, we calculated the receiving operating curve (ROC) and precision-recall curve (PRC) for the following subgroups: ER + /*HER2* + , ER + /*HER2* − , ER − /*HER2* + , ER − /*HER2* − achieving AUROCs of 0.66 ± 0.3, 0.8 ± 0.09, 0.72 ± 0.43, and 0.62 ± 0.11 (Additional File 5: Fig. 4A–H) indicating HRD could be predicted detached from morphological subtypes. Our analysis of the mutational landscape of both the HRD-H and HRD-L ground truths revealed that *TP53* had the highest alteration frequency (67%) in the HRD-H ground truth group, which was significantly greater than in the HRD-L group (20%), following alterations in the large *TTN* (26% vs. 14%) gene. In contrast, the most enriched alterations in the HRD-L subgroup were observed for the genes *PIK3CA* (39%), *CDH1* (16%), *GATA3* (14%), and *MAP3K1* (11%), whereas the prevalences of *PIK3CA*, *CDH1*,* GATA3*, and *MAP3K1* in the HRD-H subgroup were 19%, 2%, 6%, and 1%, respectively (Fig. 3C). For the HRD-H prediction subgroup, alteration frequencies for *TP53* were significantly higher at 77% (Fig. 3D). Such divergences were not as noticeable in the HRD-L prediction group. These findings imply alteration frequencies between the two subgroups differ consistently across both the ground truth and prediction data. Moreover, we compared the HRD-H prediction score to the alteration status of somatic and germline mutations in the *BRCA1/2* genes, whereupon we saw that there was a significant difference between the mutant and wild-type cases for *BRCA1* germline and *BRCA2* somatic mutations (Fig. 3E). Methylation data indicated that the HRD-H group had most of its methylation alterations in the N-shore portion of the *BRCA1* promoter region, whereas those in the HRD-L group were mainly located in the S-shore portion (Additional File 5: Fig. 4I). Lastly, we proceeded to investigate the histomorphological patterns associated with the presence of HRD through whole slide prediction heatmaps of the model trained on TCGA-UCEC and deployed on CPTAC-UCEC (Fig. 4A–C). Our findings revealed that high grade, fibrosis, hemorrhage, and lymphocytic infiltration are consistent features predictive of HRD across various tumor types, as shown in Fig. 4 for TCGA-BRCA and TCGA-UCEC, particularly in the top predicted HRD-H tiles for the top three patients in the internal cross-validation model. Fibrosis was observed in HRD-H cases, particularly in BRCA (Fig. 4D). Moreover, hemorrhagic necrosis especially adjacent to tumor tissue and tumor stroma was consistently observed as highly predictive areas in the true HRD-H cases across various cancer types and less seen in the HRD-L cases. (Additional File 6–8: Fig. 5–7). This is consistent with previously published findings where lymphocyte infiltration, fibrosis, and high tumor cell density are observed in HRD-H patients in BRCA [40]. In summary, these data show that known HRD morphology characteristics were found in our DL-based top predicted HRD-H patients.

## Discussion

HRD has recently emerged as an important biomarker for targeted treatment in solid tumors [12, 53]. However, the assessment of HRD, although better defined for patients with gynecological tumors, remains challenging in clinical routine. This, in part, can be attributed to high costs, limited availability, and the lack of comparable HRD detection methods. In this context, a pan-cancer test for HRD by DL-based image analysis of histopathology slides could be a useful pre-screening tool for the identification of HRD tumors, all the while reducing the load of genetic tests.

In this study, we demonstrated that DL can predict HRD status from histological WSIs across nine tumor types in within-cohort and external validation experiments. Interestingly, our findings revealed that a BRCA-based classifier could also detect HRD from H&E slides across diverse tumor entities. As expected, the HRD prediction was significantly lower in tumors with a low prevalence of HRD. Moreover, our classifier could also identify histomorphological characteristics such as hemorrhagic necrosis at tumor margins, lymphocyte infiltration, fibrosis, and high tumor cell density which are associated with HRD in BRCA [40]. Thus, validating the efficacy of our model. Nevertheless, despite having trained our classifier solely on BRCA, its consistent identification of HRD-associated morphological patterns across different tumor entities reiterates the value of our tool for broader applications. In contrast to previous studies, we have shown a pan-cancer DL-based prediction model consisting of a more comprehensive genomic HRD score calculated from LOH, TAI, and LST as ground truth directly from H&E tumor slides [39, 40].

Our morphological analysis revealed that UCEC or PAAD achieved better predictive results compared to LUSC or LIHC, a trend previously observed in pan-cancer studies [35, 54]. Tumors with a complex structure, such as adenocarcinomas, may be more morphologically susceptible to genetic alterations than solid tumors with a rather syncytial pattern. HRD-H tumors eventually barely resemble glandular tissue, which might be their main distinctive feature and thus a potential explanation for this constellation. Nevertheless, additional studies consisting of larger patient cohorts are needed to confirm these findings. Upon closer inspection of the TCGA-BRCA subgroups, it was revealed that predicted HRD-H is more common in triple-negative BRCA, which is known for its poor prognosis and resistance to conventional chemotherapy. In line with their ground truths, the majority of those patients were predicted to be HRD-H by our classifier (Fig. 3A, B) [52]. Furthermore, clear molecular pathological differences were found in the two subgroups. Specifically, the HRD-H subgroup is characterized by *TP53* alterations, while the HRD-L subgroup has a higher frequency of *PIK3CA* alterations, suggesting an interactive effect between the *TP53* mutated cases and HRD-H patients [55, 56]. This is particularly true for *BRCA1* mutated cancers, where HRD-H was predicted significantly better than in *BRCA1* wildtype cases (Fig. 3E) [57].

Recently, the European Medicine Agency (EMA) and FDA granted the first approval of the use of PARPi therapy for HRD-positive and *BRCA*-WT ovarian cancer patients based on the PAOLA-1 study [15, 41]. Clinical trials with promising interim data are also underway for other tumor entities and further approval is expected in the future. Despite the evident link between HRD and *BRCA1/2* mutations, it is now well established that the total number of HRD-H patients significantly exceeds the total number of *BRCA*-mutated patients in various cancer types [24, 58]. Patients who fall into this diagnostic gap can be identified with comprehensive HRD testing, as proposed in our study. These approaches, including the AI-based screening methods we have applied here, can complement *BRCA1/2* testing as a biomarker test for PARPi use. Driving diagnostic routines towards phenotype-based, rather than inconsistent molecular alteration-based HRD detection methods, might extend our ability to identify patients who may benefit from PARPi, and potentially enroll them in clinical trials. Through this proof of concept study, we have demonstrated that an HRD morphology is indeed present across diverse tumor types and can be detected through histology slides, thus potentially serving as an HRD pan-cancer marker. Prospective trials conducted in a two-step approach, where an AI-based HRD score can be evaluated for its use as a biomarker to guide treatment decisions, could potentially lead to lower sequencing requirements and cost reduction.

### Limitations

It is important to note that our study has its limitations. First, the sample sizes of our cohorts, particularly for the validation CPTAC dataset, were relatively small. This small sample size may affect the robustness and statistical power of our findings. Moreover, the variation in the distribution of HRD prevalences between tumor types can result in class imbalances. Although we applied weighing techniques during the model training process to address the effect of imbalanced datasets on the accuracy of our classifiers, this could still impact the statistical power of the results, as well as the generalisability of our models within a larger population. We observed higher AUROCs in the validation cohort, which may be attributed to the smaller size and higher class imbalance in the test set. Thus, reiterating the importance of utilizing larger patient cohorts as a requirement to validate our findings. Furthermore, the quality of the data from the TCGA and CPTAC cohorts may vary, which can also potentially impact the accuracy of our predictions. In order to implement this approach within a clinical routine setting as a pre-screening tool, further analysis with different DL models on larger datasets is necessary. Potential biases stemming from data variability and model limitations should also be addressed in future research. Future studies should extend to populations from different ethnicities such as Asian and African populations, which are likely underrepresented in this study. Moreover, due to the unavailability of germline data, we had to limit our approach by focusing solely on the use of a genomic HRD score. Lastly, we were constrained to utilize a non-cancer-specific binarization cut-off, since a consensus for clinically validated HRD cut-offs for each tumor type has yet to be developed.

## Conclusions

Our findings provide evidence that DL has the potential to not only contribute to but also improve diagnostic HRD testing. This could potentially save time and costs as well as improve patient outcomes by identifying subgroups who may benefit from targeted therapy. Current clinical practices face challenging factors such as high cost, time consumption, lack of availability, and inconsistency in HRD status screening methods. These logistic, analytic, and financial challenges contribute to the partial identification of cancer patients who may benefit from PARPi therapy and to the limited genetic testing, which is further compounded by the panoply of HRD status assessment methods whose interassay concordance is limited. With the aid of AI, we have the opportunity to identify these subgroups and improve patient outcomes. The practical implications of our findings suggest that integrating AI-driven HRD testing into clinical decision-making processes can enhance personalized medicine.

## Supplementary information

**Additional File 1:** **Figure 1.** Homologous recombination deficiency prevalences across the cohorts. (A) Overview of the total patient count (*n*=573) in the CPTAC cohort before merging the image data with the molecular data and afterward. (B) Overview of the total patient count (*n*=5,155) in the TCGA cohort before merging the image data with the molecular data and afterward. (C) Distribution of the homologous recombination deficiency high (HRD-H) and low (HRD-L) patient number among the different tumor types of The Cancer Genome Atlas (TCGA) and Clinical Proteomic Tumor Analysis Consortium (CPTAC). Abbreviations: BRCA=breast invasive carcinoma; CRC=colorectal cancer; LIHC=liver hepatocellular carcinoma; LUAD=lung adenocarcinoma; LUSC=lung squamous cell carcinoma; OV=ovarian serous cystadenocarcinoma; PAAD=pancreatic adenocarcinoma; PRAD=prostate adenocarcinoma; UCEC=uterine corpus endometrial carcinoma.

**Additional File 2:** **Table 1.** Raw statistical results. All raw experimental results related to Figure 2, including receiving operating curve (ROC) with 95% confidence interval (CI), Precision-Recall Curve (PRC) with 95% confidence interval (CI), p-values and Homologous recombination deficiency (HRD) high (HRD-H) and HRD-low (HRD-L) patient numbers based on the ground truth, for internal 5-fold cross-validation on The Cancer Genome Atlas (TCGA) external validation on Clinical Proteomic Tumor Analysis Consortium (CPTAC) for both attMIL and transformer-based approaches.. [Supplementary_Table_1_All_statistical_results.xlsx] in separate file.

**Additional File 3:** **Figure 2.** Receiving operating curve for the Internal Validation and tumor-wise external validation. The Receiving operating curve (ROC) and p-value (*p > 0.05; **p ≤ 0.05; ***p ≤ 0.01) are shown for the five-fold internal cross-validation experiments for each of the models in The Cancer Genome Atlas (TCGA) for the Homologous recombination deficiency (HRD) binary score for (A) TCGA-BRCA, (B) TCGA-CRC, (C) TCGA-LIHC, (D) TCGA-LUAD, (E) TCGA-LUSC, (F) TCGA-PAAD, (G) TCGA-PRAD, (H) TCGA-OV, (I) TCGA-UCEC; Roc curves for the external validation on the Clinical Proteomic Tumor Analysis Consortium (CPTAC) for each previously trained model for (J) CPTAC-LUAD, (K) CPTAC-LUSC, (L) CPTAC-PAAD, (M) CPTAC-UCEC. Abbreviations: BRCA=breast invasive carcinoma; CRC=colorectal cancer; LIHC=liver hepatocellular carcinoma; LUAD=lung adenocarcinoma; LUSC=lung squamous cell carcinoma; OV=ovarian serous cystadenocarcinoma; PAAD=pancreatic adenocarcinoma; PRAD=prostate adenocarcinoma; UCEC=uterine corpus endometrial carcinoma.

**Additional File 4:** **Figure 3.** Receiving operating curve for the cross-cancer external validation. The Receiving operating curve (ROC) p-value (*p > 0.05; **p ≤ 0.05; ***p ≤ 0.01) are shown for the cross-cancer external validation experiments for each model trained on The Cancer Genome Atlas (TCGA) breast cancer (BRCA) cohort for the Homologous recombination deficiency (HRD) binary score on (A) TCGA-CRC, (B) TCGA-LIHC, (C) TCGA-LUAD, (D) CPTAC-LUAD, (E) TCGA-LUSC, (F) CPTAC-LUSC, (G) TCGA-OV, (H) TCGA-PAAD, (I) CPTAC-PAAD, (J) TCGA-PRAD, (K) TCGA-UCEC, (L) CPTAC-UCEC. Abbreviations: BRCA=breast invasive carcinoma; CRC=colorectal cancer; LIHC=liver hepatocellular carcinoma; LUAD=lung adenocarcinoma; LUSC=lung squamous cell carcinoma; OV=ovarian serous cystadenocarcinoma; PAAD=pancreatic adenocarcinoma; PRAD=prostate adenocarcinoma; UCEC=uterine corpus endometrial carcinoma.

**Additional File 5:** **Figure 4. **Subgroup analysis and overview the BRCA1 promotor methylations in TCGA-BRCA. The Receiving operating curve (ROC) and Precision Recall curve (PRC) are shown for the five-fold internal cross-validation experiment for each of the models in The Cancer Genome Atlas - breast cancer (TCGA-BRCA) cohort for the Homologous recombination deficiency (HRD) score. ROC curve is represented for the four different subgroups (A) estrogen receptor positive (ER+) and HER2+ (B) ER+ and HER2- (C) ER negative (ER-) and HER2+ (D) ER- and HER2-. The PRC curve is shown for (E) ER+/HER2+, (F) ER+/HER2-, (G) ER-/HER2+, (H) ER-/HER2-. (I) Sketched representation of the occurring promotor methylations (accessed with HM27 and HM450) in the BRCA1 gene for the ground truth Homologous recombination deficiency high (HRD-H) and low (HRD-L) subgroups.

**Additional File 6:** **Figure 5.** Morphological features of Homologous recombination deficiency in breast and endometrial cancer. Whole Slide Image (WSI) and classification heatmap (ground truth: Homologous recombination deficiency high (HRD-H) and low (HRD-L) and prediction: HRD-H) with magnifications of two different regions. The model was trained on The cancer genome atlas (TCGA) breast invasive carcinoma (BRCA) cohort and deployed cross cancer wise. Top true positive predicted patients are shown for (A) TCGA-BRCA, (B) Clinical Proteomic Tumor Analysis Consortium (CPTAC) uterine corpus endometrial carcinoma (UCEC) and (C) TCGA-UCEC.

** Additional File 7:** **Figure 6.** Morphological features of Homologous recombination deficiency in pancreatic and prostate adenocarcinoma. Whole Slide Image (WSI) and classification heatmap (ground truth: Homologous recombination deficiency high (HRD-H) and low (HRD-L) and prediction: HRD-H) with magnifications of two different regions. The model was trained on The cancer genome atlas (TCGA) breast invasive carcinoma (BRCA) cohort and deployed cross cancer wise. Top true positive predicted patients are shown for (A) TCGA pancreatic adenocarcinoma (PAAD), (B) Clinical Proteomic Tumor Analysis Consortium (CPTAC) pancreatic adenocarcinoma (PAAD) and (C) TCGA prostate adenocarcinoma (PRAD).

**Additional File 8:** **Figure 7.** Comparison of Homologous recombination deficient and proficient tissue slides. Whole Slide Images (WSIs) comparing Homologous recombination deficient high (HRD-H) and low (HRD-L) patients in three different tumor types of The cancer genome atlas (TCGA). (A) TCGA. BRCA HRD-H, (B) TCGA- BRCA HRD-L, (C) TCGA - UCEC HRD-H, (D) TCGA-UCEC HRD-L, (E) TCGA-PRAD HRD-H, (F) TCGA-PRAD HRD-L. Abbreviation: breast invasive carcinoma (BRCA), uterine corpus endometrial carcinoma (UCEC), prostate adenocarcinoma (PRAD).

**Additional File 9:** **Table 2.** Homologous recombination deficiency score Tables. Training data and calculated homologous recombination deficiency score (HRD) out of the three subscores loss of heterozygosity (LOH), telomeric allelic imbalance (TAI) and large-scale state transitions (LST) available as continuous (HRDsum) and binary (HRD_Binary) target with a chosen cut-off of HRD-L=42 for patients of The Cancer Genome Atlas (TCGA, Sheet1) and Clinical Proteomic Tumor Analysis Consortium (CPTAC, Sheet2).

**Additional File 10:** **Table 3.** Weblink for customized Homologous recombination deficiency (HRD) subgroups. Weblink for accessing the clinical and molecular characteristics in both the ground truth and prediction Homologous recombination Deficiency (HRD) subgroups at www.cbioportal.org for The Cancer Genome Atlas breast cancer (TCGA-BRCA) Pan Cancer Atlas 2018 study and the TCGA-BRCA Firehose Legacy cohort.

## Data Availability

The WSI, molecular, and clinical data for the TCGA and CPTAC cohorts are publicly available at https://portal.gdc.cancer.gov/ and https://www.cbioportal.org/ (accessed, 08 March 2022). The script used for calculating the HRD score is available at https://github.com/sztup/scarHRD (accessed 06 June 2022). All other source codes can be downloaded under https://github.com/KatherLab/marugoto. Our calculated HRD score is publicly available in Additional File 9: Table 2. Moreover, our custom TCGA-BRCA HRD-H and HRD-L group can be accessed for the PanCancer Atlas cohort at https://www.cbioportal.org/ (Additional File 10: Table 3).

## Abbreviations

AI: Artificial intelligence
ASCAT: Allele-Specific Copy Number Analysis of Tumors
attMIL: Attention-weighted multiple instance learning
AUPRC: Area under the precision-recall curve
AUROC: Area under the receiver operating characteristic curve
BRCA: Breast invasive carcinoma
BRCA1/2: Breast cancer genes 1 and 2
CI: Confidence interval
CIOMS: Council for International Organizations of Medical Sciences
CPTAC: Clinical Proteomic Tumor Analysis Consortium
CRC: Colorectal adenocarcinoma
DL: Deep learning
DSB: DNA double-strand breaks
EMA: European Medicine Agency
ER-: Estrogen receptor negative
ER +: Estrogen receptor positive
FDA: U.S. Food and Drug Administration
GDC: Genomic Data Commons
GIS: Genomic instability score
H&E: Hematoxylin and eosin
HR: Homologous recombination
HRD-H: HRD high
HRD-L: HRD low
HRD: Homologous recombination deficiency
HRR: Homologous recombination repair
LIHC: Liver hepatocellular carcinoma
LOH: Loss of heterozygosity
LST: Large-scale state transition
LUAD: Lung adenocarcinoma
LUSC: Lung squamous cell carcinoma
OV: Ovarian serous cystadenocarcinoma
PAAD: Pancreatic adenocarcinoma
PARP: Poly(ADP-Ribose)-polymerase
PARPi: Poly(ADP-Ribose)-polymerase inhibitor
PRAD: Prostate adenocarcinoma
PRC: Precision-recall curve
ROC: Receiving operating curve
SBS3: Single base substitution 3
SNP: Single nucleotide polymorphism
SSDBs: Single strand DNA breaks
SSL: Self-supervised learning
TAI: Telomeric allelic imbalance
TCGA: The Cancer Genome Atlas
TRIPOD: Transparent reporting of a multivariable prediction model for individual prognosis or diagnosis
UCEC: Uterine corpus endometrial carcinoma
WSI: Whole slide image
WSIs: Whole slide images
