# Supplementary figures and images for: Prediction of homologous recombination deficiency from routine histology with attention-based multiple instance learning in nine different tumor types
Source: BMC Biol. 2024 Oct 8;22:225. doi: 10.1186/s12915-024-02022-9 (PMC11462727; doi:10.1186/s12915-024-02022-9)

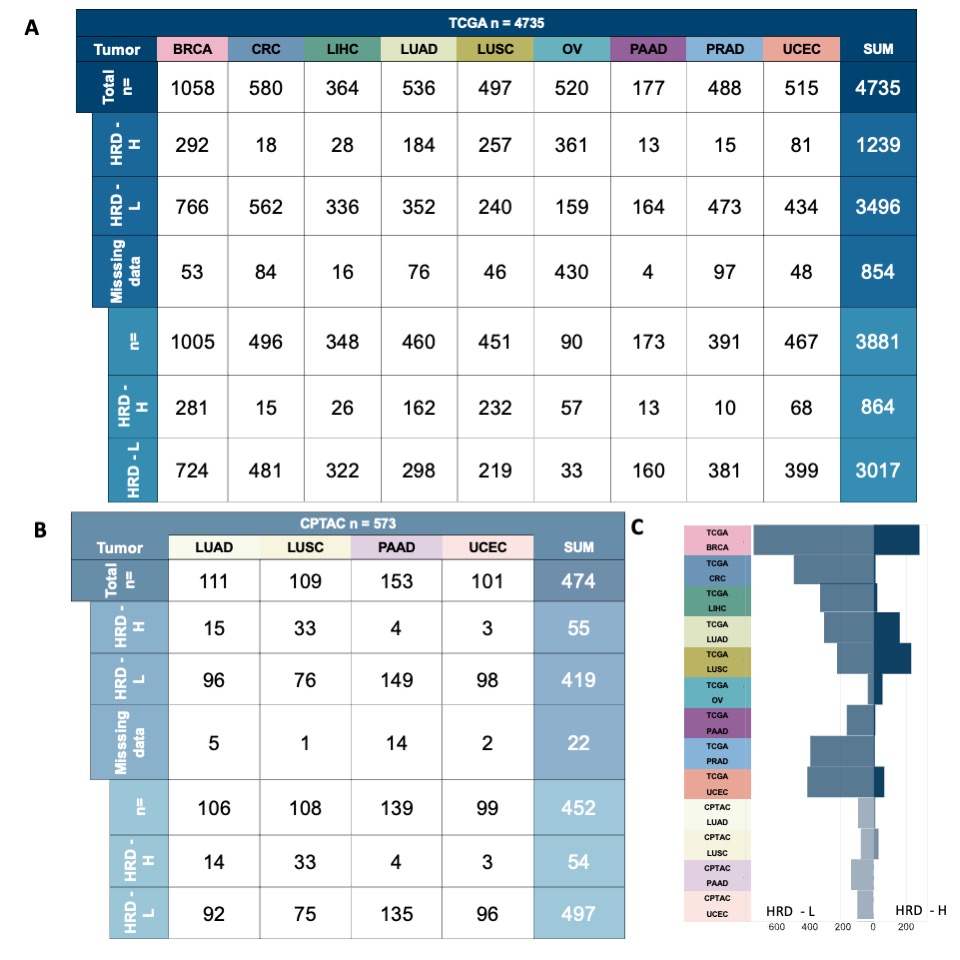

Supplement: Supplementary file 1 — Additional File 1: Figure 1. Homologous recombination deficiency prevalences across the cohorts. (A) Overview of the total patient count (n=573) in the CPTAC cohort before merging the image data with the molecular data and afterward. (B) Overview of the total patient count (n=5,155) in the TCGA cohort before merging the image data with the molecular data and afterward. (C) Distribution of the homologous recombination deficiency high (HRD-H) and low (HRD-L) patient number among the different tumor types of The Cancer Genome Atlas (TCGA) and Clinical Proteomic Tumor Analysis Consortium (CPTAC). Abbreviations: BRCA=breast invasive carcinoma; CRC=colorectal cancer; LIHC=liver hepatocellular carcinoma; LUAD=lung adenocarcinoma; LUSC=lung squamous cell carcinoma; OV=ovarian serous cystadenocarcinoma; PAAD=pancreatic adenocarcinoma; PRAD=prostate adenocarcinoma; UCEC=uterine corpus endometrial carcinoma. [file 12915_2024_2022_MOESM1_ESM.jpeg]

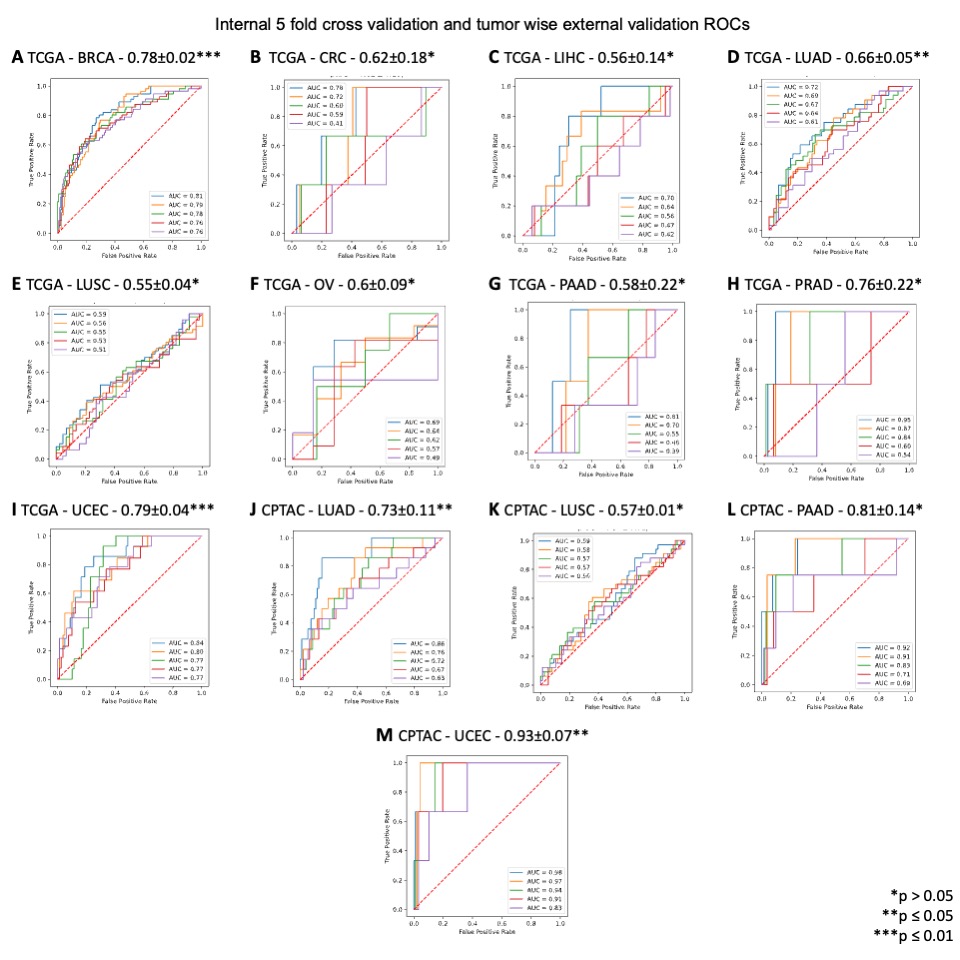

Supplement: Supplementary file 3 — Additional File 3: Figure 2. Receiving operating curve for the Internal Validation and tumor-wise external validation. The Receiving operating curve (ROC) and p-value (*p > 0.05; **p ≤ 0.05; ***p ≤ 0.01) are shown for the five-fold internal cross-validation experiments for each of the models in The Cancer Genome Atlas (TCGA) for the Homologous recombination deficiency (HRD) binary score for (A) TCGA-BRCA, (B) TCGA-CRC, (C) TCGA-LIHC, (D) TCGA-LUAD, (E) TCGA-LUSC, (F) TCGA-PAAD, (G) TCGA-PRAD, (H) TCGA-OV, (I) TCGA-UCEC; Roc curves for the external validation on the Clinical Proteomic Tumor Analysis Consortium (CPTAC) for each previously trained model for (J) CPTAC-LUAD, (K) CPTAC-LUSC, (L) CPTAC-PAAD, (M) CPTAC-UCEC. Abbreviations: BRCA=breast invasive carcinoma; CRC=colorectal cancer; LIHC=liver hepatocellular carcinoma; LUAD=lung adenocarcinoma; LUSC=lung squamous cell carcinoma; OV=ovarian serous cystadenocarcinoma; PAAD=pancreatic adenocarcinoma; PRAD=prostate adenocarcinoma; UCEC=uterine corpus endometrial carcinoma. [file 12915_2024_2022_MOESM3_ESM.jpeg]

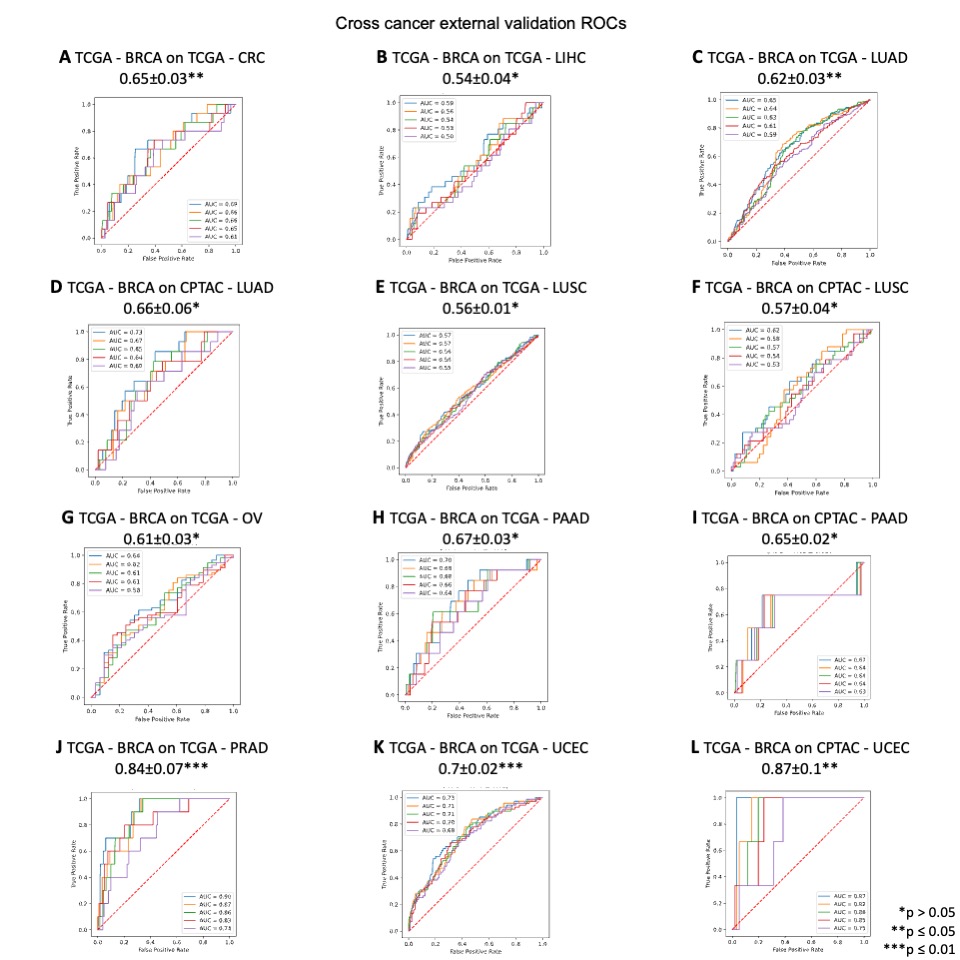

Supplement: Supplementary file 4 — Additional File 4: Figure 3. Receiving operating curve for the cross-cancer external validation. The Receiving operating curve (ROC) p-value (*p > 0.05; **p ≤ 0.05; ***p ≤ 0.01) are shown for the cross-cancer external validation experiments for each model trained on The Cancer Genome Atlas (TCGA) breast cancer (BRCA) cohort for the Homologous recombination deficiency (HRD) binary score on (A) TCGA-CRC, (B) TCGA-LIHC, (C) TCGA-LUAD, (D) CPTAC-LUAD, (E) TCGA-LUSC, (F) CPTAC-LUSC, (G) TCGA-OV, (H) TCGA-PAAD, (I) CPTAC-PAAD, (J) TCGA-PRAD, (K) TCGA-UCEC, (L) CPTAC-UCEC. Abbreviations: BRCA=breast invasive carcinoma; CRC=colorectal cancer; LIHC=liver hepatocellular carcinoma; LUAD=lung adenocarcinoma; LUSC=lung squamous cell carcinoma; OV=ovarian serous cystadenocarcinoma; PAAD=pancreatic adenocarcinoma; PRAD=prostate adenocarcinoma; UCEC=uterine corpus endometrial carcinoma. [file 12915_2024_2022_MOESM4_ESM.jpeg]

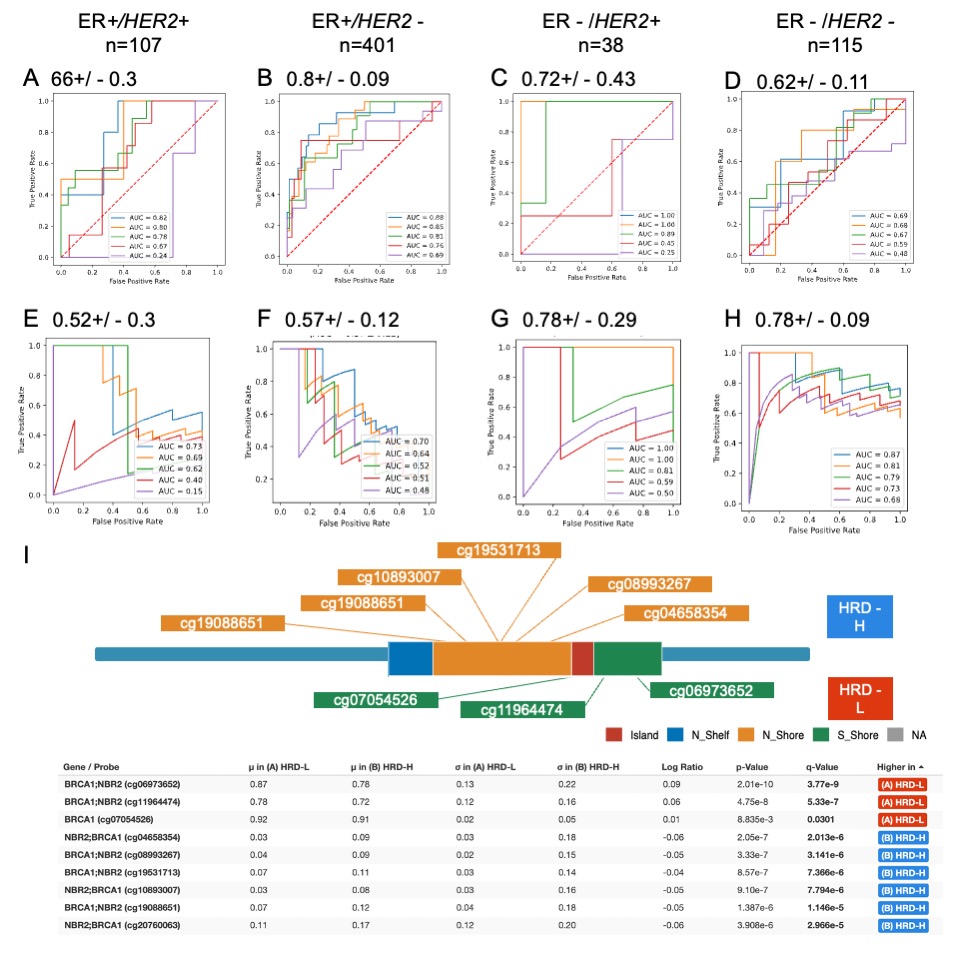

Supplement: Supplementary file 5 — Additional File 5: Figure 4. Subgroup analysis and overview the BRCA1 promotor methylations in TCGA-BRCA. The Receiving operating curve (ROC) and Precision Recall curve (PRC) are shown for the five-fold internal cross-validation experiment for each of the models in The Cancer Genome Atlas - breast cancer (TCGA-BRCA) cohort for the Homologous recombination deficiency (HRD) score. ROC curve is represented for the four different subgroups (A) estrogen receptor positive (ER+) and HER2+ (B) ER+ and HER2- (C) ER negative (ER-) and HER2+ (D) ER- and HER2-. The PRC curve is shown for (E) ER+/HER2+, (F) ER+/HER2-, (G) ER-/HER2+, (H) ER-/HER2-. (I) Sketched representation of the occurring promotor methylations (accessed with HM27 and HM450) in the BRCA1 gene for the ground truth Homologous recombination deficiency high (HRD-H) and low (HRD-L) subgroups. [file 12915_2024_2022_MOESM5_ESM.jpeg]

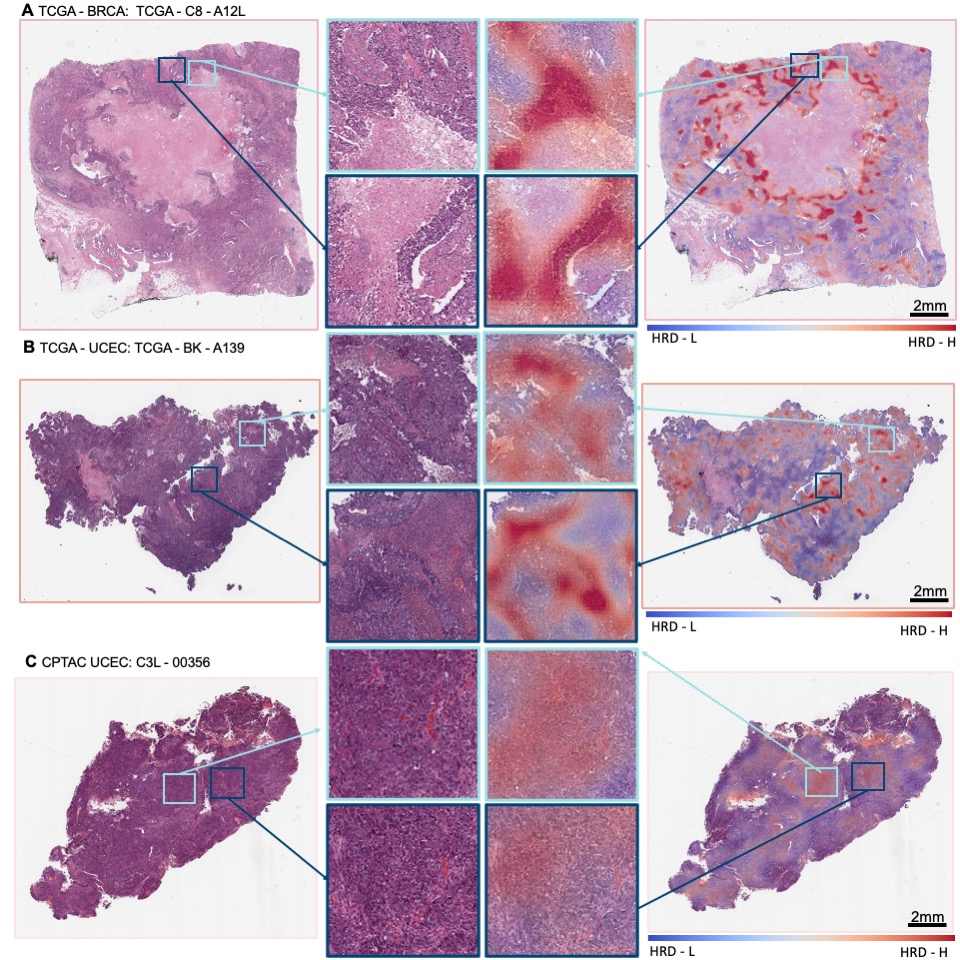

Supplement: Supplementary file 6 — Additional File 6: Figure 5. Morphological features of Homologous recombination deficiency in breast and endometrial cancer. Whole Slide Image (WSI) and classification heatmap (ground truth: Homologous recombination deficiency high (HRD-H) and low (HRD-L) and prediction: HRD-H) with magnifications of two different regions. The model was trained on The cancer genome atlas (TCGA) breast invasive carcinoma (BRCA) cohort and deployed cross cancer wise. Top true positive predicted patients are shown for (A) TCGA-BRCA, (B) Clinical Proteomic Tumor Analysis Consortium (CPTAC) uterine corpus endometrial carcinoma (UCEC) and (C) TCGA-UCEC. [file 12915_2024_2022_MOESM6_ESM.jpeg]

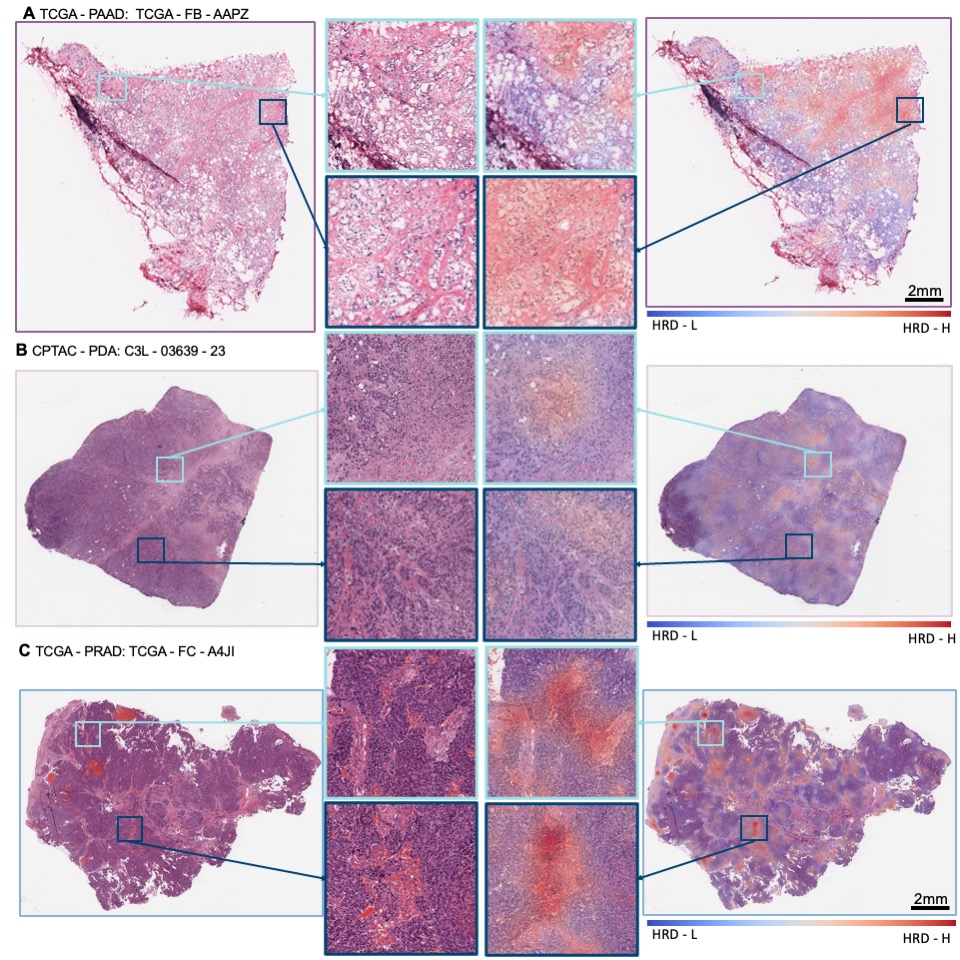

Supplement: Supplementary file 7 — Additional File 7: Figure 6. Morphological features of Homologous recombination deficiency in pancreatic and prostate adenocarcinoma. Whole Slide Image (WSI) and classification heatmap (ground truth: Homologous recombination deficiency high (HRD-H) and low (HRD-L) and prediction: HRD-H) with magnifications of two different regions. The model was trained on The cancer genome atlas (TCGA) breast invasive carcinoma (BRCA) cohort and deployed cross cancer wise. Top true positive predicted patients are shown for (A) TCGA pancreatic adenocarcinoma (PAAD), (B) Clinical Proteomic Tumor Analysis Consortium (CPTAC) pancreatic adenocarcinoma (PAAD) and (C) TCGA prostate adenocarcinoma (PRAD). [file 12915_2024_2022_MOESM7_ESM.jpeg]

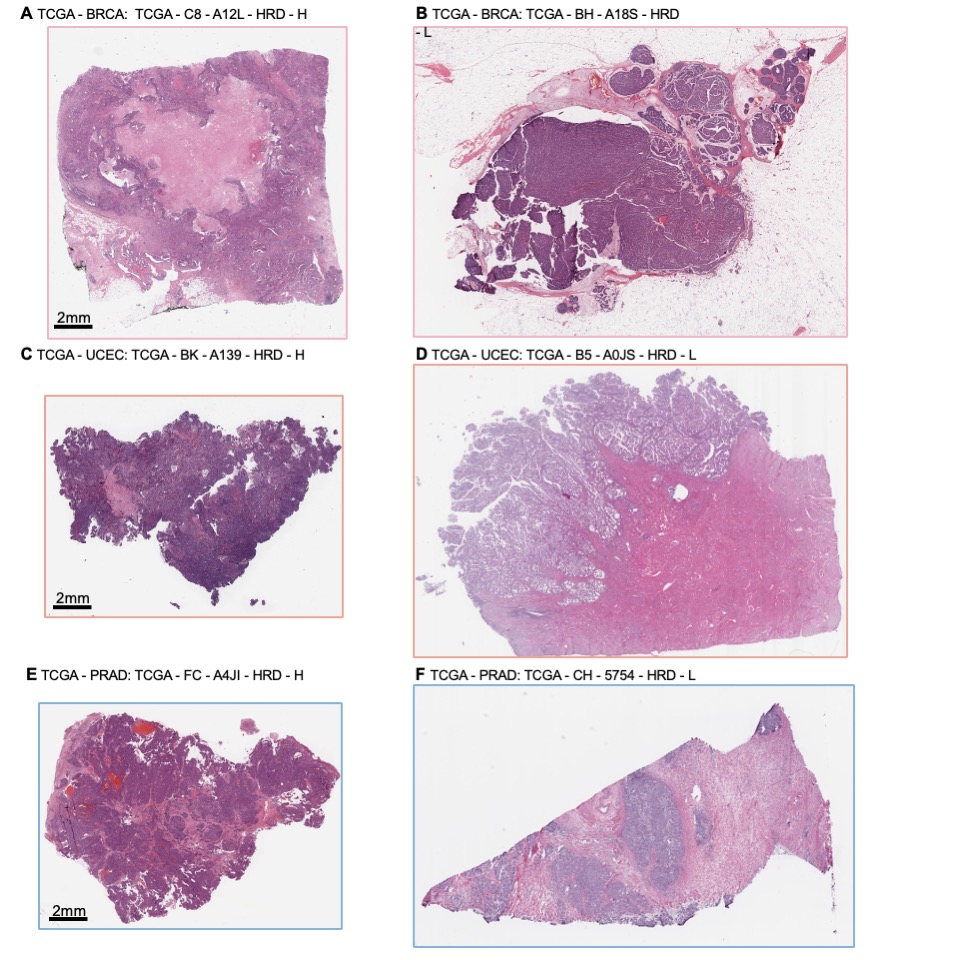

Supplement: Supplementary file 8 — Additional File 8: Figure 7. Comparison of Homologous recombination deficient and proficient tissue slides. Whole Slide Images (WSIs) comparing Homologous recombination deficient high (HRD-H) and low (HRD-L) patients in three different tumor types of The cancer genome atlas (TCGA). (A) TCGA. BRCA HRD-H, (B) TCGA- BRCA HRD-L, (C) TCGA - UCEC HRD-H, (D) TCGA-UCEC HRD-L, (E) TCGA-PRAD HRD-H, (F) TCGA-PRAD HRD-L. Abbreviation: breast invasive carcinoma (BRCA), uterine corpus endometrial carcinoma (UCEC), prostate adenocarcinoma (PRAD). [file 12915_2024_2022_MOESM8_ESM.jpg]
