# Supplementary material for: Prediction of homologous recombination deficiency from routine histology with attention-based multiple instance learning in nine different tumor types
Source: BMC Biol. 2024 Oct 8;22:225. doi: 10.1186/s12915-024-02022-9 (PMC11462727; doi:10.1186/s12915-024-02022-9)
Supplement: Supplementary file 10 — Additional File 10: Table 3. Weblink for customized Homologous recombination deficiency (HRD) subgroups. Weblink for accessing the clinical and molecular characteristics in both the ground truth and prediction Homologous recombination Deficiency (HRD) subgroups at www.cbioportal.org for The Cancer Genome Atlas breast cancer (TCGA-BRCA) Pan Cancer Atlas 2018 study and the TCGA-BRCA Firehose Legacy cohort. [file 12915_2024_2022_MOESM10_ESM.docx]

Supplementary Table 3

Weblink for accessing the clinical and molecular characteristics for both the customized ground truth and prediction Homologous recombination Deficiency (HRD) subgroups at [www.cbioportal.org](http://www.cbioportal.org) for

1. The Cancer genome Atlas breast cancer (TCGA-BRCA) Pan Cancer Atlas 2018 study

<https://www.cbioportal.org/study/summary?id=brca_tcga_pan_can_atlas_2018#sharedGroups=63e612daabb2dd578e28faf7,63e612e41cec6922c423553a,63eba36d1cec6922c423630e,63eba37c1cec6922c423630f>

1. TCGA-BRCA Firehose Legacy cohort

<https://www.cbioportal.org/study/summary?id=brca_tcga#sharedGroups=63e6129eabb2dd578e28faf5,63e612af1cec6922c4235538>
